# Effects of different traditional Chinese exercise in the treatment of essential hypertension: a systematic review and network meta-analysis

**DOI:** 10.3389/fcvm.2024.1300319

**Published:** 2024-02-28

**Authors:** Qingyuan Zhang, Xiaogang Xu, Qianyan Wu, Jingwen Zhang, Shenghe Huang, Lin Wu, Minping Tian, Delin Zhang

**Affiliations:** ^1^ College of Traditional Chinese Medicine and College of Life Sciences, Jiangxi University of Chinese Medicine, Nanchang, Jiangxi, China; ^2^ Evidence-Based Medicine Research Center, Jiangxi University of Chinese Medicine, Nanchang, Jiangxi, China; ^3^ School of Clinical Medicine, Jiangxi University of Chinese Medicine, Nanchang, Jiangxi, China; ^4^ Institute of Traditional Chinese Medicine and Health Development, Jiangxi University of Chinese Medicine, Nanchang, Jiangxi, China

**Keywords:** essential hypertension, traditional Chinese exercise, Tai Chi, Baduanjin, systematic review, network meta-analysis

## Abstract

**Background:**

As a therapy to prevent and treat essential hypertension (EH), traditional Chinese exercises (TCEs) were widely used in clinical practice. However, there is a lack of strictly comparison of the antihypertensive efficacy of different TCEs, which not conducive to the selection of the best and most optimal treatment. This study aimed to perform a network meta-analysis to objectively evaluate which TCE has the best effects in assisting with lowering blood pressure.

**Methods:**

PubMed, Embase, the Cochrane Library, Chinese National Knowledge Infrastructure (CNKI), VIP, SinoMed and Wanfang Data were searched for all randomized controlled trials (RCTs) on TCEs for the treatment of EH published up to July 10, 2023. RoB2.0 tool was utilized to evaluate the quality of the RCTs. The network meta-analysis was performed by R 4.1.2 and Stata 17.0. Weighted mean difference (WMD) was calculated for continuous outcomes.

**Results:**

A total of 29 studies, including 2,268 patients were included to analyze 6 different interventions. The network meta-analysis results presented that in comparison with control group, Tai Chi + antihypertensive medication [WMD = −10.18, 95% CI, (−14.94, −5.44)] is the most effective intervention for lowering systolic blood pressure (SBP), and Wuqinxi + antihypertensive medication [WMD = −10.36, 95% CI (−18.98, −1.66)] is the most effective intervention for lowering diastolic blood pressure (DBP).

**Conclusion:**

TCEs combined with antihypertensive medication may be able to achieve more prominent antihypertensive effects with Tai Chi and Wuqinxi potentially being the higher-priority options. However, well-designed randomized studies are warranted to further verify currently conclusion.

## 1 Introduction

Hypertension is a clinical syndrome characterized by increased systemic arterial pressure and increased peripheral arteriolar resistance. According to epidemiological investigation, the number of people aged 30–79 years with hypertension doubled from 1990 to 2019, and the number of people with hypertension is expected to reach 1.56 billion (1.54–1.58 billion) worldwide by 2025 (1, 2). As compared with healthy people, the risk of cardiovascular and cerebrovascular diseases in hypertensive patients is considerably greater (3, 4). Anti-hypertension medication still serves as a major therapy choice for treating hypertension now. However, due to medication side effects, treatment resistance as well as economic factors, an extensive number of patients still have hypertension that cannot be successfully controlled (5, 6). Therefore, it is essential to further explore a widely available, low-cost and sustainable treatment method (7).

Traditional Chinese exercises (TCEs) are an aerobic exercise therapy that focuses on regulating breathing and controlling the mind (8), which mainly includes Tai Chi (TC), Baduanjin (BDJ), Liuzijue (LZJ) and Wuqinxi (WQX). Presently, the total number of people practicing TCEs worldwide has surpassed 6.5 million across 56 countries or regions (9). Due to their ease of adoption, widespread availability, and affordability, TCEs have been utilized as an adjunct therapy in treating various cardiovascular and cerebrovascular diseases in China (10–13). Several systematic reviews and meta-analysis have evaluated the efficacy of TCEs in the treatment of hypertension (14–16). However, their conclusions are all based on a pairwise meta-analysis which is limited in the fact that they can only compare the efficacy of paired interventions. In addition, some kinds of TCEs (Liuzijue, Wuqinxi) were neglected and not included in the evaluation, which not conducive to the selection of the best and most optimal treatment. Therefore, a systematic and comprehensive study is urgently required to effectively compare and rank the antihypertensive effects of the different TCEs.

Different from the traditional meta-analysis, network meta-analysis (NMA) can efficiently compare up to three or more interventions at the same time (17), which allows the investigators to compare and rank the effectiveness of multiple interventions directly or indirectly (18, 19). This study aims to utilized Bayesian network meta-analysis method, which accounts for both direct and indirect comparisons to provide valid evidence supporting the application of TCEs for EH.

## 2 Methods

This network meta-analysis was reported in accordance with the Preferred Reporting Items for Systematic Reviews and Meta-analysis 2020 (PRISMA 2020) (20), and the PRISMA checklist can be located in Supplementary Material S1. The protocol of this review was registered in International prospective register of systematic reviews (PROSPERO) under registration number CRD42023438688.

### 2.1 Databases and retrieval strategy

A combination of manual and computer retrieval methods was utilized and the retrieval databases include CNKI, Wanfang Data, VIP, SinoMed, PubMed, Cochrane Library, and Embase. The search was for randomized controlled trials (RCTs) of different TCEs for the treatment of EH. The last retrieval date was July 10, 2023, and all relevant retrieval strategies of the database were listed in Supplementary Material S2.

### 2.2 Inclusion criteria

The inclusion criteria for this study was strictly adhered to the PICOS framework, and the inclusion criteria are as follows: (1) Type of study: published randomized controlled trials (RCTs) for the treatment of essential hypertension; (2) Patients: patients that were diagnosed with essential hypertension with systolic blood pressure (SBP) ≥ 140 mmHg and/or diastolic blood pressure (DBP) ≥ 90 mmHg; (3) Interventions: the treatment group received TCEs combined with conventional antihypertensive medication; (4) Comparison: the control group received conventional antihypertensive medication or conventional antihypertensive medication combined with aerobic exercise; (5) Outcomes: changes in SBP and DBP after treatment (blood pressure change = prior treatment blood pressure value−post-treatment blood pressure value).

### 2.3 Exclusion criteria

The exclusion criteria are as follows: (1) The patients were diagnosed with secondary hypertension; (2) Duplicated publications; (3) Studies that failed to reported the baseline which results in an inability to calculate changes in SBP and DBP after treatment; (4) Studies that only reported antihypertensive efficiency rate but failed to provide specific numerical values.

### 2.4 Study selection and data extraction

EndNote X9 was used to complete the selection process of the studies. Firstly, removed the duplicates. Subsequently, two reviewers (WQY and HSH) independently read through the titles, abstracts as well as full texts meticulously. At the same time, the reviewers strictly adhered to the inclusion criteria and exclusion criteria to screening the literature. If the event when a disagreement occurred between the two reviewers, we would refer to the views of the third researcher (WL).

After the screening process, the data for final inclusion in the literature were extracted by two reviewers (WL and TMP) using a pre-designed data extraction form that consists of the following: (1) Basic information of literature that includes the title, first author, corresponding author, year, intervention, comparison, as well as the methods of random sequence generation and allocation concealment; (2) Basic information of the patients which includes the gender and age; (3) Information of intervention which includes the intensity, frequency, and duration; (4) SBP and DBP values before and after treatment. Lastly, any disagreement that arose in the data extraction process shall be resolved through a team discussion.

### 2.5 Risk of bias assessment

The risk of bias for the included trials was independently evaluated by the two researchers (ZQY and XXG) using the Revised Cochrane Risk of Bias Tool (RoB 2.0) (21). The evaluation contents predominantly include (1) randomization process; (2) deviations from intended interventions; (3) missing outcome data; (4) measurement of the outcome; (5) selection of the reported result. To assess the risk of bias within each domain, one or more signaling questions were answered, which generally contain five alternative answers: (1) yes; (2) probably yes; (3) no information; (4) probably no; (5) no. Based on reviewers' responses to the signal questions, the risk of bias for each domain can be rated on three levels, respectively as low risk of bias, some concerns and high risk of bias. Subsequently, judgments within each domain contributed to an overall risk of bias assessment per study. Any discrepancy in the assessment of RoB2 would be discussed to reach a consensus.

### 2.6 Statistical analysis

The systolic and diastolic blood pressures are the outcomes in this study. The difference between the baseline and endpoint of systolic and diastolic blood pressures were calculated and included in the analysis in order to determine the best and most optimal treatment for hypertension more accurately and intuitively. The calculation formula is as follows (22):

$$SD_{\mathrm{change}}=\sqrt{{\mathrm{SD}}_{\mathrm{baseline}}^{2}+{\mathrm{SD}}_{\mathrm{final}}^{2}-(2\times \mathrm{Corr}\times SD_{\mathrm{baseline}}\times SD_{\mathrm{final}})}$$

The included data were presented as the weighted mean difference (WMD) with a 95% confidence interval (CI). The network evidence plots were generated utilizing Stata17.0 software. Bayesian random-effects Network Meta-Analysis (NMA) for all outcomes was performed using R4.1.2 with the gemtc package (23, 24). The initial setup included four Markov chains, a step size of 1, 20,000 pre-iterations for burn-in, and 50,000 iterations to achieve convergence. Following iterations for convergence, a PSRF nearing 1indicated satisfactory model convergence; otherwise, iterations were extended. When there was a closed loop, the node splitting method was utilized to test the local inconsistency, and it was judged that the consistency was good and favorable when *P* > 0.05 (25). Stata17.0 software was employed to generate surface under the cumulative ranking curve (SUCRA) plots for effective intervention ranking and comparison-adjusted funnel plots. Begg's test was conducted using R4.1.2 with the netmeta package. Subsequently, by combining funnel plots with Begg's test, we examined whether there was publication bias in the NMA. Finally, the I^2^ statistic assessed heterogeneity, the robustness of the results of this study were assessed by including treatment duration as a covariate to conduct a meta-regression analysis.

### 2.7 Evidence quality evaluation

For each outcome, the quality of evidence across the network was assessed using the Confidence in Network Meta-Analysis (CINeMA) framework that is based on the Grading of Recommendations Assessment, Development, and Evaluation (GRADE) approach (26, 27). The evaluation contents included within-study bias, reporting bias, indirectness, imprecision, heterogeneity, and incoherence. In the CINeMA framework, the confidence rating is an overall qualitative assessment that summarizes judgment across the 6 domains which assess the quality of evidence. Similarly, being identical to the GRADE framework, confidence can be classified as high, moderate, low, or very low (28).

## 3 Results

### 3.1 Study selection

**Figure 1:**
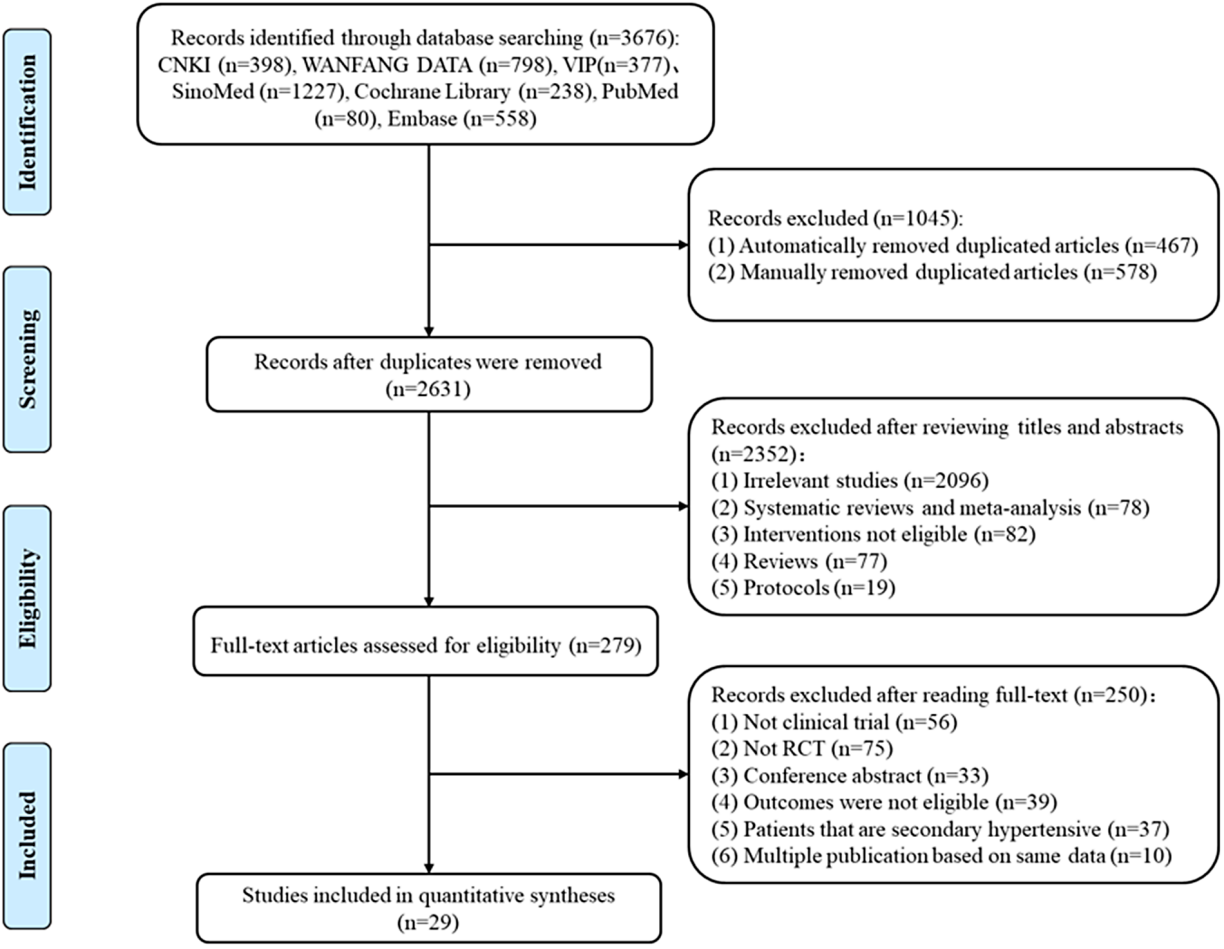
Flow of the selection process for included studies.

A total of 3,676 articles were initially identified. Subsequently, through meticulous reading and screening of each respective records, 29 studies (29–57) were eventually included in the network meta-analysis, and no additional studies were identified by screening the reference lists of the relevant reviews and selected studies. The process of inclusion and exclusion was presented in Figure 1.

### 3.2 Basic characteristics of the included literature

A total of 2,268 patients with 1,155 in the treatment group and 1,133 in the control group in the included 29 clinical studies completed the trial. The shortest course of treatment in treatment group for TCEs was 8 weeks, and the longest course of treatment was 6 months. At the same time, the most common practice frequency was 1–2 times/day, and the duration of a single practice was mostly maintained at 30–60 min. The basic characteristics of included patients and studies were listed in the Supplementary Material S3.

### 3.3 Assessment of risk of bias

**Figure 2:**
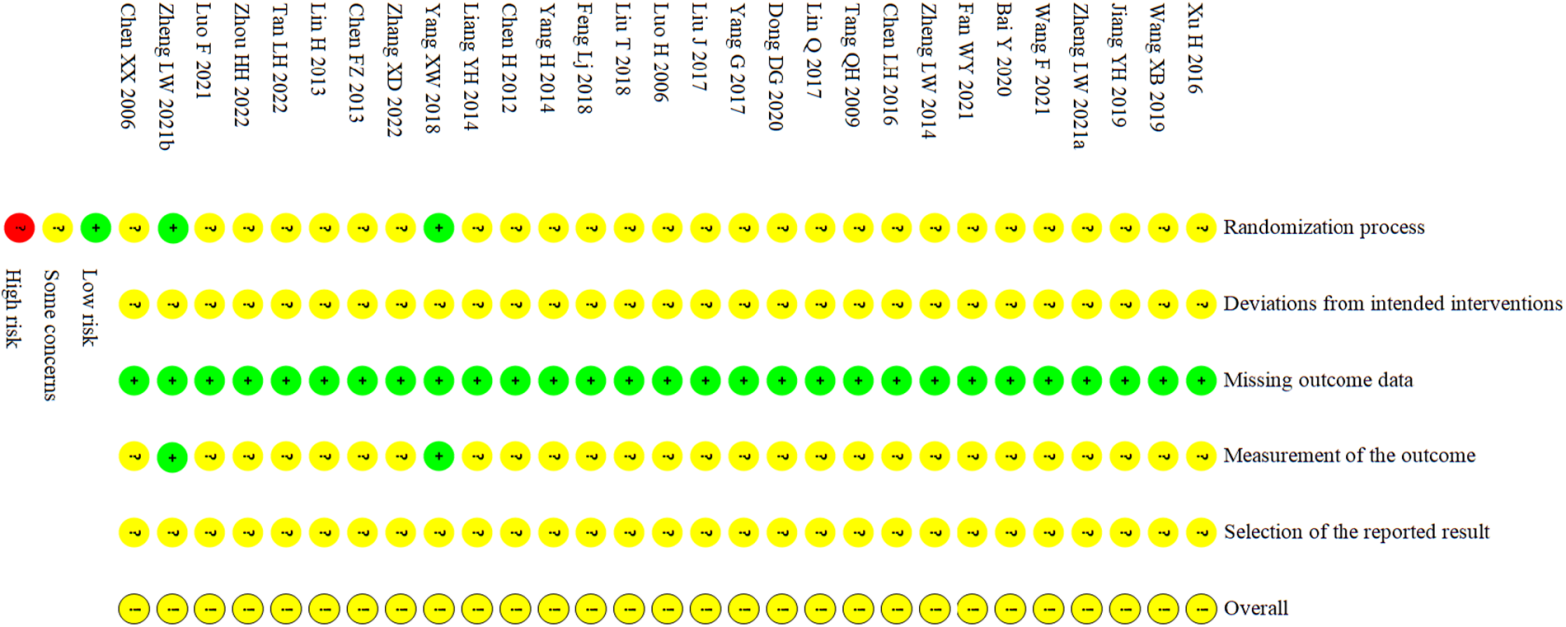
Risk of bias assessment of included studies.

In terms of the randomization process, two studies (49, 56) detailed reported the methods of randomized grouping and used the sealed opaque containers to conceal the allocation. Meanwhile, there was no observed differences between intervention groups at baseline, so these studies were assessed as low risk of bias, and the remaining studies were assessed as moderate risk of bias cause they did not indicate whether allocation concealment was performed or failed to report the specific method of randomization. In terms of deviations from intended interventions, all studies presented a moderate risk of bias due to the peculiarity of the interventions made it difficult to perfectly perform blinding to patients and carers. In terms of missing outcome data, all studies were assessed as low risk of bias due to the absence of significant withdrawal as well as the adoption of appropriate data analysis methods. In terms of outcome measurement, two studies (49, 56) were assessed as low risk of bias due to the application of appropriate outcome assessment methods and blinded the outcome assessors, while the remaining studies were assessed as moderate risk of bias. As all studies were unable to attain their trial protocols, the risks for selective reporting were all assessed as moderate risks of bias. Lastly, for the overall risk of bias, all studies were assessed as moderate risk of bias. The detail of the assessment was showed in Figure 2.

### 3.4 Network evidence plot and model construction

**Figure 3:**
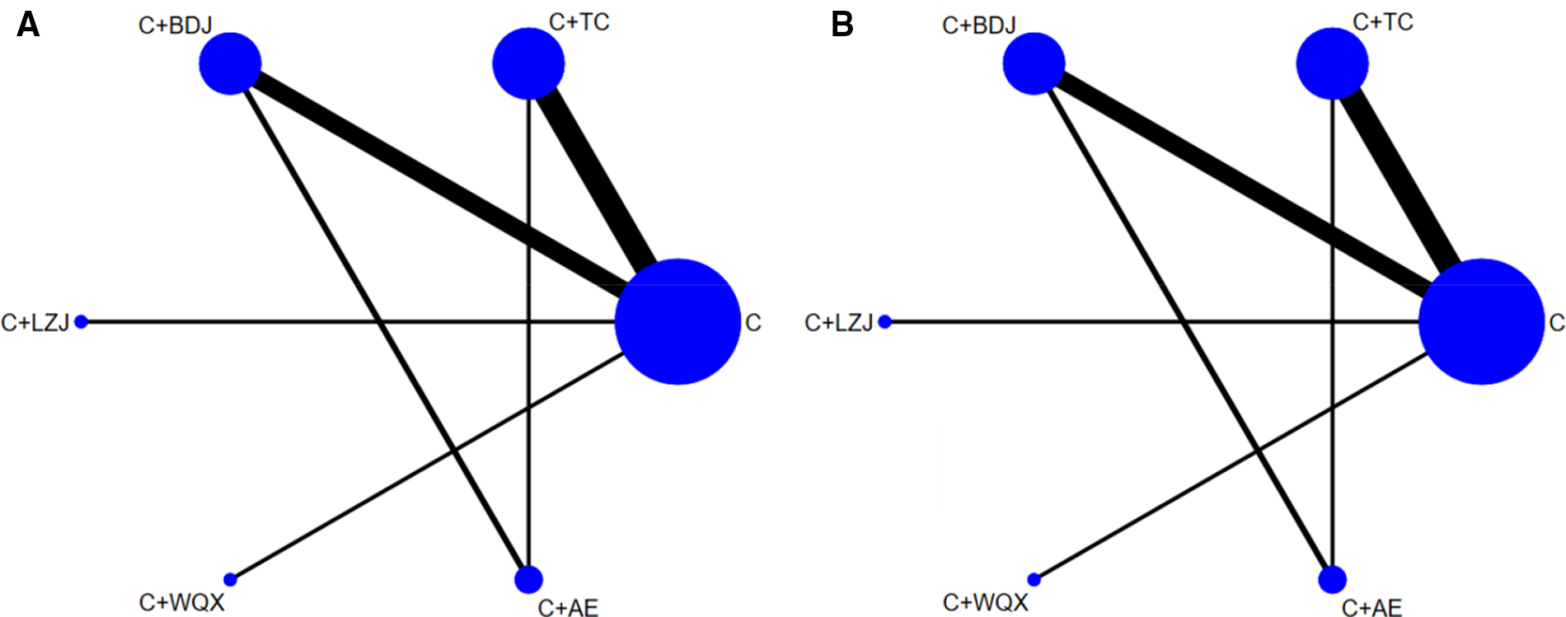
(**A**) The network plot of systolic blood pressure; (**B**) the network plot of diastolic blood pressure. C, conventional antihypertensive medication; TC, Tai Chi; BDJ, Baduanjin; LZJ, Liuzijue; WQX, Wuqinxi; AE, aerobic exercise.

A total of 29 studies (29–57) with 6 interventions were included in the network meta-analysis. The network plots were presented in Figure 3. The inconsistency test demonstrated that the deviance information criterion (DIC) difference between the consistent model and the inconsistent model was less than 5 which indicates good consistency. Therefore, the consistent model was selected for the network meta-analysis.

### 3.5 Network meta-analysis

#### 3.5.1 Systolic blood pressure

A total of 29 studies (29–57) reported the results of the SBP. The network meta-analysis presented that conventional antihypertensive medication + Tai Chi [WMD = −10.18, 95% CI, (−14.94, −5.44)], conventional antihypertensive medication + Baduanjin [WMD = −7.61, 95% CI (−12.88, −2.31)] were significantly better as compared to use conventional antihypertensive medication alone in improving SBP. However, there was no statistical significance observed between the other interventions as presented in the Table 1, and the heterogeneity test between the studies was showed in Supplementary Material S4.

**Table 1:** Pairwise comparison of different interventions for lowering SBP.

C					
−**10.18** (−**14.94,**	** **	** **	** **	** **	** **
−5.44)*	C + TC				
−**7.61** (−**12.88,** −2.31)*	2.58 (−4.17, 9.32)	C + BDJ			
−7.24 (−19.00, 4.44)	2.95 (−9.71, 15.59)	0.37 (−12.52, 13.21)	C + LZJ		
−9.83 (−21.56, 1.86)	0.34 (−12.3, 12.95)	−2.25 (−15.12, 10.61)	−2.59 (−19.22, 13.98)	C + WQX	
−1.02 (−9.56, 7.55)	9.17 (0.55, 17.75)	6.59 (−1.48, 14.73)	6.24 (−8.25, 20.7)	8.82 (−5.66, 23.39)	C + AE

The black bold font represents that the comparison between interventions are statistically significant.

C, conventional antihypertensive medication; TC, Tai Chi; BDJ, Baduanjin; LZJ, Liuzijue; WQX, Wuqinxi; AE, aerobic exercise.

*The comparison between interventions are statistically significant.

#### 3.5.2 Diastolic blood pressure

A total of 29 studies (29–57) reported the results of the DBP. The results of the network meta-analysis presented that compared to conventional antihypertensive medication, conventional antihypertensive medication + Tai Chi [WMD = −5.99, 95% CI (−9.39, −2.57)], conventional antihypertensive medication + Baduanjin [WMD = −6.93, 95% CI (−10.79, −3.04)] and conventional antihypertensive medication + Wuqinxi [WMD = −10.36, 95% CI (−18.98, −1.66)] were significantly better in lowering DBP. Lastly, there was no statistical difference observed between the other treatments as presented in the Table 2, and the heterogeneity test between the studies was showed in Supplementary Material S4.

**Table 2:** Pairwise comparison of different interventions for lowering DBP.

C					
−**5.99** (−9.39, −2.57)*	C + TC				
−**6.93** (−10.79, −3.04)*	−0.95 (−5.84, 3.97)	C + BDJ			
−8.02 (−16.61, 0.65)	−2.04 (−11.31, 7.30)	−1.09 (−10.50, 8.43)	C + LZJ		
−**10.36** (−**18.98,** −1.66)*	−4.37 (−13.64, 4.98)	−3.43 (−12.91, 6.13)	−2.33 (−14.59, 9.92)	C + WQX	
−3.04 (−9.24, 3.17)	2.94 (−3.28, 9.24)	3.90 (−2.05, 9.82)	4.98 (−5.69, 15.60)	7.32 (−3.38, 17.94)	C + AE

The black bold font represents that the comparison between interventions are statistically significant.

C, conventional antihypertensive medication; TC, Tai Chi; BDJ, Baduanjin; LZJ, Liuzijue; WQX, Wuqinxi; AE, aerobic exercise.

*The comparison between interventions are statistically significant.

#### 3.5.3 Ranking results of efficacy

**Figure 4:**
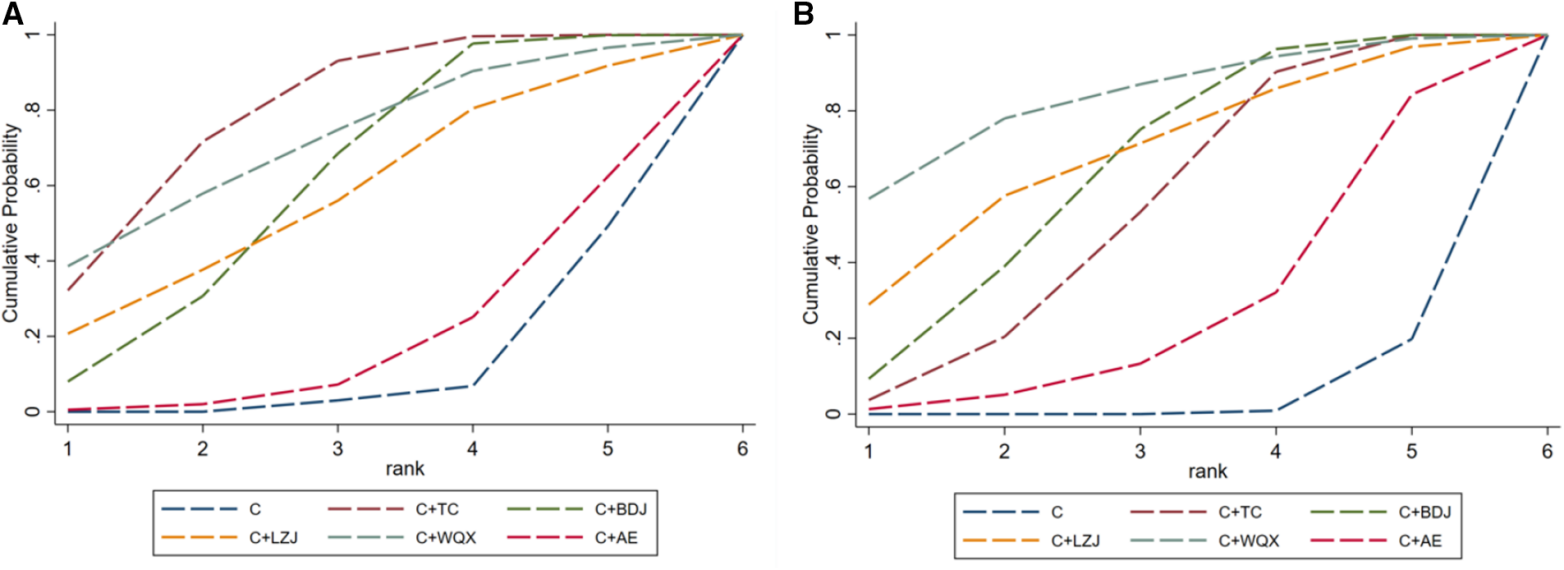
(**A**) The probability ranking of different TCEs in lowering SBP; (**B**) the probability ranking of different TCEs in lowering DBP. C, conventional antihypertensive medication; TC, Tai Chi; BDJ, Baduanjin; LZJ, Liuzijue; WQX, Wuqinxi; AE, aerobic exercise.

We ranked all the interventions involved according to the results calculated by SUCRA. The therapeutic effect of different interventions in lowering SBP is in descending order as follows: C + TC > C + WQX > C + BDJ > C + LZJ > C + C + AE > C, as shown in Figure 4A. Correspondingly, the therapeutic effect of different interventions in lowering DBP is in descending order as follows: C + WQX > C + LZJ > C + BDJ > C + TC > C + AE > C, as shown in Figure 4B.

#### 3.5.4 Meta-regression analysis

Considering the variations in the duration and frequency of exercises among the included studies, we performed a meta-regression analysis to assess their potential impact on the antihypertensive effect. The results of the meta-regression analysis indicated that beyond an 8-week period of TCEs, the duration of treatment might not be the primary factor influencing the therapeutic effect. However, the varying frequency of TCEs may affect the antihypertensive effect. The results of the meta-regression were listed in the Supplementary Material S5.

### 3.6 Certainty assessment of evidence

A total of 15 treatment comparisons were evaluated, the confidence ratings of the treatment comparisons were judged as rated as very low to moderate. The main reasons for downgrading including within-study bias, indirectness and heterogeneity. The details of evidence assessment were presented in the Supplementary Material S6.

### 3.7 Safety assessment

After a thorough and meticulous review of the included articles, one study (32) evaluated on its safeness and it is reported to exhibit zero adverse events.

### 3.8 Inconsistency test and convergence analysis

The evidence network plots of SBP and DBP both have closed loops. Therefore, a local inconsistency test was required. The results of node splitting portrayed that there was no significant difference between the direct and indirect comparisons of the network meta-analysis for systolic and diastolic blood pressure (*P* > 0.05), indicating that the consistency is good. The diagnostic results of convergence showed that the model test of all network meta-analyses was 1.00 ≤ PSRF ≤ 1.05, and the analysis results were reliable. The results of inconsistency test and convergence analysis can be located in the Supplementary Material S7.

### 3.9 Publication bias

**Figure 5:**
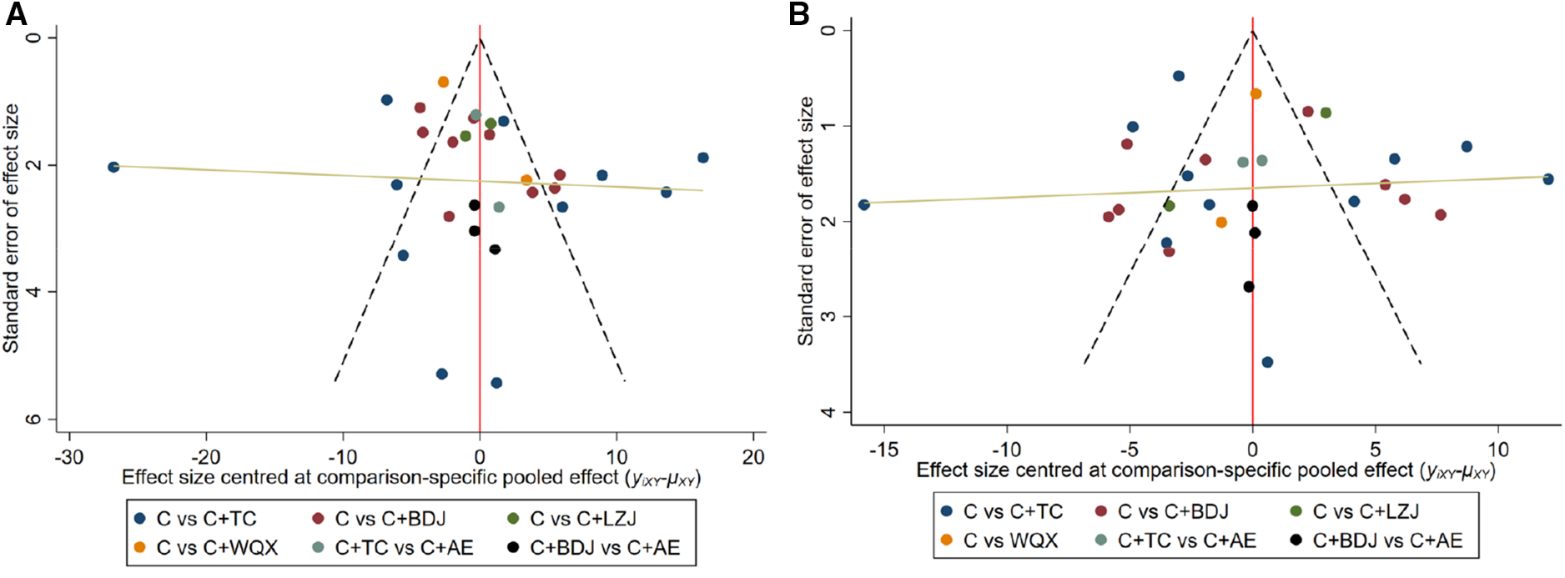
(**A**) Bias specific pooled effect for SBP; (**B**) bias specific pooled effect for DBP. C, conventional antihypertensive medication; TC, Tai Chi; BDJ, Baduanjin; LZJ, Liuzijue; WQX, Wuqinxi; AE, aerobic exercise.

The comparison-adjusted funnel plots showed that all the studies were basically distributed on both sides of the midline, as shown in Figure 5. While Begg's test (*P* = 0.4531 for SBP, *P* = 0.5994 for DBP) indicated no observable publication bias (Supplementary Material S8). From the results, it could be considered that there was no significant publication bias.

## 4 Discussion

### 4.1 Summary of main finding

Large-scale epidemiological studies have provided definitive evidence that high blood pressure maintains a continuous graded association with the risk of fatal and nonfatal stroke, ischemic heart disease, heart failure, and noncardiac vascular disease (58, 59). Although regular aerobic exercise can lower blood pressure, due to the high physical fitness requirements of aerobic exercise, most adults with hypertension are unwilling or unable to exercise. Therefore, many alternative therapies for aerobic exercise, such as Tai Chi, Baduanjin, Wuqinxi and Liuzijue (60–62), have been widely recommended in clinical practice and proved to be effective. The purpose of this study was to compare which TCE possesses the best effects on essential hypertension. In this network meta-analysis, we included 29 studies that involved 2,268 participants and compared the antihypertensive effects of 6 interventions. Our results showed that in lowering blood pressure, C + TC, C + WQX, C + BDJ, C + LZJ, C + AE resulted in better outcomes for patients with essential hypertension compared with the C group. Based on the probability ranking, it is clear that C + TC is the most effective intervention for improve SBP. Regarding the improvement of DBP, C + WQX is the most effective intervention.

Tai Chi is not solely aerobic; it also encompasses muscle-strengthening elements, especially for the quadriceps. Research has demonstrated that Tai Chi results in significant improvements in handgrip strength, functional capacity, postural balance, and thoracolumbar spine flexibility (63). During Tai Chi practice, subjects in a meditative state try to shift their weight slowly from side to side while concurrently concentrating and visualizing each movement sequence. This process aids in efficient muscle utilization, enhancing circulation, and regulating the autonomic control of blood pressure (64). Wuqinxi is a traditional Chinese health practice attributed to Hua Tuo, a renowned ancient Chinese physician. It involves a series of bio-mimetic techniques emulating the movements of tigers, deer, bears, apes, and birds. Similar to Tai Chi, during Wuqinxi practice, attaining a state of mind and body that is “relaxed, tranquil, natural, and harmonious internally and externally” is essential (65). These attributes potentially contribute to the effectiveness of both Tai Chi and Wuqinxi in lowering blood pressure.

In our NMA, we found high heterogeneity in some directly pairwise comparison (C VS C + TC and C VS C + BDJ), considering that the therapeutic effect of TCEs may be related to the course or frequency of treatment (66), we conducted a meta-regression analysis to explore the potential impact of these two covariates. The meta-regression results showed that while the duration of treatment might not be the primary factor influencing the therapeutic effect, the varying frequency of TCEs may affect the antihypertensive effect. Due to the limitations of the number of included articles and the quality of the study, the meta-regression results should not be interpreted excessively. Although duration of treatment might not be the primary factor influencing the therapeutic effect, we still recommend that in future clinical applications, more comprehensive consideration should be given to the duration of individual sessions and the total duration of treatment in order to avoid the situation where a favorable effect cannot be successfully attained due to a short exercise time or that the physical strength and exercise enthusiasm of patients are affected by a pro-long exercise duration. Moreover, the frequency of exercises and duration of single exercise sessions has also varied considerably in clinical design among studies. Taking all these factors into account, the certainty of evidence was rated as ranging from very low to moderate due to methodological deficiencies and imprecision among studies, but this review presents an updated overview of different TCEs for essential hypertension.

### 4.2 Mechanisms

Although the mechanism of TCEs in treating hypertension is not clear, the increase in endogenous nitric oxide (NO) production and the decrease in endothelin-1 (ET-1) in plasma after TCEs therapy may be one of the main biological mechanisms of TCEs in treating essential hypertension (36). As a potent vasodilator factor, NO can induce vasodilation, improve microvascular reactivity, and increase blood perfusion. Therefore, through the intervention by TCEs, the blood flow in patients was redistributed, and the blood flow in the skeletal muscle and myocardium will be increased correspondingly, and this will lead to an increase in shear stress. Simultaneously, the increase of vascular shear stress will also stimulate the release of NO from the blood vessels and stimulate the activity of nitric oxide synthase at the same time. In this case, it will further promote the production of NO to increase, thus achieving the aim of relaxing blood vessels and lowering blood pressure (56, 67, 68). In contrast to NO, ET-1 is a powerful vasoconstrictor, it affects salt and water homeostasis by affecting the renin-angiotensin-aldosterone system and vasopressin, thereby increasing blood pressure (69). Animal study showed that appropriate physical activity will reduce the ET levels, while excessive exercise will increase plasma ET levels correspondingly (70), as a mind-body exercise, TCEs have an appropriate amount of physical activity. Therefore, after the intervention of TCEs, the hypertension of patients is controlled correspondingly under the combination of increased NO production and decreased ET-1 content. Furthermore, as TCEs belong to a type of aerobic exercise that is guided by the mind, patients will be able to effectively relieve tensions and promote aerobic metabolism of the whole body through long-term practice. By acting on the cerebral cortex and vasomotor center, TCEs will be able to regulate the body's functional state, increases the tension of the vagus nerve, decreases the excitability of the sympathetic nerve, promotes vasorelaxation, and contributes to a significant role in lowering blood pressure by reducing peripheral vascular resistance (71, 72).

### 4.3 Study advantages and limitations

Compared to previous study (73), we have the following advantages. First, we registered the protocol before our work began and we used explicit inclusion and exclusion criteria to ensure the similarity between included studies which reduced the risk of bias of this NMA. Second, we firstly provided the evidence of Liuzijue in the treatment of EH. Third, we used the CINeMA tool to evaluate the certainty of evidence and provided the level of evidence for each comparison. Finally, according to the nodal split model, it is clear that there is no significant difference between direct or indirect comparisons for each split node (*P* > 0.05), while the diagnostic plots for each outcome showed that the median and 97.5% values of the contraction factor converge to 1, which indicates that our findings are stable and reliable.

Our study was not without its limitations. First, all included studies had moderate bias risks. Second, the differences in sample size, baseline, frequency of practice and age of the patients inevitably contributed to existence of clinical heterogeneity. Third, since there are relatively few clinical studies that use Wuqinxi or Liuzijue as interventions, there may be a small sample size effect which may exaggerate their therapeutic effect. Finally, most of the evidence provided in this study is based on indirect comparisons, more direct comparative evidence a required in the future to verify the current conclusions.

## 5 Conclusion

In summary, TCEs combined with antihypertensive medication may be able to achieve more prominent antihypertensive effects with Tai Chi and Wuqinxi potentially being the higher-priority options. However, it should be highlighted that, because of the low level of evidence of most of the included studies we cannot make any strong suggestions, until more well-designed randomized studies with large sample sizes are conducted.
