# Supplementary material for: Effects of different traditional Chinese exercise in the treatment of essential hypertension: a systematic review and network meta-analysis
Source: Front Cardiovasc Med. 2024 Feb 28;11:1300319. doi: 10.3389/fcvm.2024.1300319 (PMC10935740; doi:10.3389/fcvm.2024.1300319)
Supplement: Supplementary file 1 [file Datasheet1.zip › Supplementary material 2.docx]

**Supplementary materials 2**

The search strategy

| **Database** | **NO.** | **Search terms** |
| --- | --- | --- |
| **PubMed** | **#1** | (((((hypertension[MeSH Terms]) OR (hypertension[Title/Abstract])) OR (blood pressure[Title/Abstract])) OR (essential hypertension[MeSH Terms])) OR (essential hypertension[Title/Abstract])) OR (blood pressure[MeSH Terms]) |
|  | **#2** | (((((((((((((Tai Chi[MeSH Terms]) OR (Tai Ji[MeSH Terms])) OR (Tai Chi[Title/Abstract])) OR (Tai Ji[Title/Abstract])) OR (Qigong[MeSH Terms])) OR (Qigong[Title/Abstract])) OR (Baduanjin[Title/Abstract])) OR (Yijinjing[Title/Abstract])) OR (Wuqinxi[Title/Abstract])) OR (Liuzijue[Title/Abstract])) OR (Six-character formula[Title/Abstract])) OR (Eight-section brocade[Title/Abstract])) OR (Twelve-section brocade[Title/Abstract])) OR (Tao Yin[Title/Abstract]) |
|  | **#3** | #1 AND #2 Filters: **Clinical Trial, Randomized Controlled Trial** |
| **Cochrane Library** | **#1** | MeSH descriptor: [Hypertension] explode all trees |
|  | **#2** | MeSH descriptor: [Essential Hypertension] explode all trees |
|  | **#3** | MeSH descriptor: [Blood Pressure] explode all trees |
|  | **#4** | ((hypertension OR essential hypertension OR blood pressure OR high blood pressure)):ti,ab,kw |
|  | **#5** | #1 or #2 or #3 or #4 |
|  | **#6** | MeSH descriptor: [Tai Ji] explode all trees |
|  | **#7** | MeSH descriptor: [Qigong] explode all trees |
|  | **#8** | ((Tai Chi OR Tai Ji OR blood Baduanjin OR Wuqinxi OR Liuzijue OR Six-character formula) OR Eight-section brocade OR Twelve-section brocade OR Tao Yin):ti,ab,kw |
|  | **#9** | #6 OR #7 OR #8 |
|  | **#10** | #5 AND #9 |
| **Embase** | **#1** | 'hypertension'/exp OR 'essential hypertension'/exp OR 'blood pressure'/exp OR hypertension:ti,ab,kw OR 'essential hypertension':ti,ab,kw OR 'blood pressure':ti,ab,kw |
|  | **#2** | 'tai chi'/exp OR 'tai chi':ti,ab,kw OR 'tai ji'/exp OR 'tai ji':ti,ab,kw OR 'qigong'/exp OR qigong:ti,ab,kw OR baduanjin:ti,ab,kw OR yijinjing:ti,ab,kw OR liuzijue:ti,ab,kw OR 'six-character formula':ti,ab,kw OR 'eight-section brocade':ti,ab,kw OR 'twelve-section brocade':ti,ab,kw OR 'tao yin':ti,ab,kw |
|  | **#3** | #1 AND #2 AND [clinical study]/lim |
| **CNKI** | **# 1** | SU=高血压+降压+血压异常+血压 |
|  | **# 2** | 太极拳+八段锦+易筋经+五禽戏+六字诀+十二段锦+导引养生功+马王堆导引术+太极养生杖+导引术 |
|  | **# 3** | # 1 AND # 2 |
| **WANFANG DATA** | **# 1** | 主题:("血压" or "高血压" or "降压" or "血压异常") |
|  | **# 2** | ("太极拳" or "八段锦" or "易筋经" or "五禽戏" or "六字诀" or "十二段锦" or "导引养生功" or "马王堆导引术" or "太极养生杖" or "导引术") |
|  | **# 3** | #1 AND #2 |
| **VIP** | **# 1** | M=(高血压 OR 降压 OR 血压异常 OR 血压) |
|  | **# 2** | (太极拳 OR 八段锦 OR 易筋经 OR 五禽戏 OR 六字诀 OR 十二段锦 OR 导引养生功 OR 马王堆导引术 OR 太极养生杖 OR 气功 OR 导引术) |
|  | **# 3** | #1 AND #2 |
| **SinoMed** | **# 1** | ("高血压"[常用字段:智能]) |
|  | **# 2** | ("太极拳"[常用字段:智能] OR "八段锦"[常用字段:智能] OR "气功"[常用字段:智能] OR "五禽戏"[常用字段:智能] OR "易筋经"[常用字段:智能] OR "六字诀"[常用字段:智能] OR "十二段锦"[常用字段:智能] OR "导引养生功"[常用字段:智能] OR "马王堆导引术"[常用字段:智能] OR "太极养生杖"[常用字段:智能] OR "导引"[常用字段]:智能) |
|  | **# 3** | #1 AND #2 |
