# Supplementary material for: Effects of different traditional Chinese exercise in the treatment of essential hypertension: a systematic review and network meta-analysis
Source: Front Cardiovasc Med. 2024 Feb 28;11:1300319. doi: 10.3389/fcvm.2024.1300319 (PMC10935740; doi:10.3389/fcvm.2024.1300319)
Supplement: Supplementary file 1 [file Datasheet1.zip › Supplementary material 3.docx]

**Supplementary materials 3**

| **Study** | **Interventions** | | **Participants** | | **Gender(male/female)** | | **Age: mean ± SD or range** | | **Course of**  **treatment** | **Assess manner of blood pressure** |
| --- | --- | --- | --- | --- | --- | --- | --- | --- | --- | --- |
|  | **T** | **C** | **T** | **C** | **T** | **C** | **T** | **C** |  |  |
| Xu H 2016 | C+TC | C | 30 | 30 | 17/15 | 45273 | 38.07±8.09 | 37.63±9.09 | 8W | Casual blood pressure |
| Wang XB & Ye LP 2019 | C+TC | C | 50 | 50 | 20/30 | 22/28 | 67.6±4.5 | 67.4±4.2 | 3M | Casual blood pressure |
| Jiang YH et al. 2019 | C+BDJ | C | 50 | 50 | 25/25 | 26/24 | 64.67±3.15 | 65.23±3.23 | 12W | Casual blood pressure |
| Zheng LW et al. 2021 | C+BDJ | C | 33 | 34 | 23/15 | 17/21 | - | - | 12W | Casual blood pressure |
| Wang F & Wang H 2021 | C+BDJ | C | 30 | 30 | 17/13 | 45249 | 60.28±3.73 | 59.17±3.26 | 3M | Casual blood pressure |
| Bai Y et al. 2020 | C+BDJ | C | 76 | 76 | 42/34 | 40/36 | 65.96±4.98 | 66.14±5.02 | 24W | Casual blood pressure |
| Fan WY et al. 2021 | C+BDJ | C | 38 | 38 | 21/17 | 19/19 | 71.87±0.76 | 71.95±2.88 | 12W | Casual blood pressure |
| Zheng LW et al. 2014 | C+BDJ | C | 27 | 28 | 13/14 | 45276 | 69.23±3.72 | 70.06±4.36 | 12W | Casual blood pressure |
| Chen LH 2016 | C+BDJ | C | 28 | 28 | 15/13 | 14/14 | 69.98±3.11 | 70.29±1.77 | 12W | Casual blood pressure |
| Tang QH 2009 | C+TC | C | 16 | 16 | 9/9 | 10/6 | - | | 6M | Casual blood pressure |
| Lin Q & Yan XH 2017 | C+BDJ | C | 58 | 58 | - | - | - | | 6M | Casual blood pressure |
| Dong DG et al. 2020 | C+BDJ | C | 23 | 24 | 13/10 | 13/11 | - | | 16W | Casual blood pressure |
| Yang G 2017 | C+LZJ | C | 20 | 20 | 9/11 | 8/12 | 58.10±6.95 | 57.70±6.30 | 12W | Casual blood pressure |
| Liu J 2017 | C+TC | C | 80 | 77 | 47/33 | 48/29 | 43.00±6.57 | 42.60±5.67 | 24W | Casual blood pressure |
| Luo H 2006 | C+TC | C | 44 | 40 | 24/20 | 21/19 | 44.74±12.10 | 44.86±13.05 | 6M | Casual blood pressure |
| Liu T et al. 2018 | C+TC | C | 35 | 35 | 18/17 | 19/16 | 62.4±2.4 | 63.1±2.1 | 6M | Ambulatory blood pressure |
| Feng LJ et al. 2018 | C+TC | C+AE | 36 | 37 | 19/17 | 14/23 | 66.33±4.74 | 67.51±4.09 | 12W | Casual blood pressure |
| Yang H et al. 2014 | C+BDJ | C+AE | 35 | 35 | 13/22 | 19/16 | 60.07±5.84 | 60.60±7.37 | 24W | Casual blood pressure |
| Chen H & Zhou YN 2012 | C+BDJ | C+AE | 40 | 40 | 25/15 | 23/17 | 59±6 | 60±5 | 24W | Casual blood pressure |
| Liang YH et al. 2014 | C+BDJ | C+AE | 30 | 30 | 20/10 | 18/12 | 54.8±7.6 | 55.7±8.8 | 6M | Casual blood pressure |
| Yang XW 2018 | C+TC | C+AE | 37 | 38 | 16/21 | 18/20 | 66.59±4.53 | 67.47±5.12 | 16W | Casual blood pressure |
| Zhang XD et al. 2022 | C+LZJ | C | 50 | 50 | 15/35 | 17/33 | 57.50±10.16 | 58.00±10.03 | 3M | Casual blood pressure |
| Chen FZ & Lv QB 2013 | C+TC | C | 50 | 18 | - | | 30～82 | | 12W | Casual blood pressure |
| Lin H & Huang SJ 2013 | C+WQX | C | 68 | 59 | - | | 60～ | | 6M | Ambulatory blood pressure |
| Tan LH et al. 2022 | C+BDJ | C | 42 | 42 | 22/20 | 19/23 | 68.93±7.21 | 68.95±7.21 | 12W | Casual blood pressure |
| Zhou HH 2022 | C+WQX | C | 43 | 43 | 21/22 | 20/23 | 35～70 | 31～70 | 3M | Casual blood pressure |
| Luo F 2021 | C+BDJ | C | 40 | 40 | 24/16 | 27/13 | 66.54±10.32 | 67.32±9.46 | 12W | Casual blood pressure |
| Zheng LW et al. 2021 | C+BDJ | C | 26 | 27 | 13/18 | 15/16 | 45～75 | 45～75 | 24W | Casual blood pressure |
| Chen XX & Lv HQ 2006 | C+TC | C | 20 | 20 | 9/11 | 13/7 | 64.3 | 60.7 | 10W | Casual blood pressure |

| **Study** | **Details of intervention in the control group** | **Details of exercise in the treatment group** |
| --- | --- | --- |
|  |  |  |
| Xu H 2016 | Conventional antihypertensive medication | Tai Chi exercise, twice-daily, 15 mins per session |
| Wang XB & Ye LP 2019 | Conventional antihypertensive medication | Tai Chi exercise, 3 times/week, 40-60 mins per session |
| Jiang YH et al. 2019 | Amlodipine tablets, 5mg qd; flupentixol and melitracen tablets, 0.5~1 tablets qd | Baduanjin exercise, twice-daily |
| Zheng LW et al. 2021 | Conventional antihypertensive medication | Baduanjin exercise, twice-daily, 5d/week, 13 mins per session |
| Wang F & Wang H 2021 | Amlodipine tablets, 5mg qd | Baduanjin exercise, once-daily, 5d/week, 30 mins per session |
| Bai Y et al. 2020 | Telmisartan tablets, 5mg qd; diltiazem hydrochloride sustaind-release capsules | Baduanjin exercise, twice-daily, 5d/week, 45 mins per session |
| Fan WY et al. 2021 | Lekadipine tablets, 10mg qd; losartan Potassium, 10mg qd | Baduanjin exercise, twice-daily, 5d/week, 13 mins per session |
| Zheng LW et al. 2014 | Amlodipine besylate tablets qd, 5mg; telmisartan, 80mg qd | Baduanjin exercise, once-daily, 5d/week, 30 mins per session |
| Chen LH 2016 | Conventional antihypertensive medication | Baduanjin exercise, 5 times/week, 1 h per session |
| Tang QH 2009 | Conventional antihypertensive medication | Tai Chi exercise, 3-5 times/week, 30-60 mins per session |
| Lin Q & Yan XH 2017 | Amlodipine besylate, 5mg qd; telmisartan, 80mg qd | Baduanjin exercise, once-daily, 30-40 mins per session |
| Dong DG et al. 2020 | Amlodipine besylate, 5mg qd; telmisartan, 80mg qd | Baduanjin exercise, twice-daily, 5 times/week, 60 mins per session |
| Yang G 2017 | Conventional antihypertensive medication | Liuzijue exercise, 4 times/week, 1.5 hrs per session |
| Liu J 2017 | Conventional antihypertensive medication | Tai Chi exercise, twice-daily, 40 mins per session |
| Luo H 2006 | Cilazapril tablets, 2.5~5mg qd | Tai Chi exercise, once-daily, 45 mins per session |
| Liu T et al. 2018 | Cilazapril tablets, 2.5~5mg qd | Tai Chi exercise, once-daily, 1hr per session |
| Feng LJ et al. 2018 | Conventional antihypertensive medication | Tai Chi exercise, 3 times/week, 1hr per session |
| Yang H et al. 2014 | Conventional antihypertensive medication | Baduanjin exercise, 5 times/week, 40 mins per session |
| Chen H & Zhou YN 2012 | Nifedipine tablets, 10~20mg, bid | Baduanjin exercise, twice-daily, 20 mins per session |
| Liang YH et al. 2014 | Conventional antihypertensive medication | Baduanjin exercise, twice-daily, 20 mins per session |
| Yang XW 2018 | Conventional antihypertensive medication | Tai Chi exercise, 3 times/week, 1hr per session |
| Zhang XD et al. 2022 | Amlodipine besylate, 5mg qd | Liuzijue exercise, 5 times/week, 40 mins per session |
| Chen FZ & Lv QB 2013 | Conventional antihypertensive medication | Tai Chi exercise, 6 times/week, 30 mins per session |
| Lin H & Huang SJ 2013 | Conventional antihypertensive medication | Wuqinxi exercise, not less than 6 times/week, 30 mins per session |
| Tan LH et al. 2022 | Valsartan capsules, 80mg qd; eszolam tablets, 1mg qd | Baduanjin exercise, 5-7 times/week, 20-30 mins per session |
| Zhou HH 2022 | Conventional antihypertensive medication | Wuqinxi exercise, not less than 5 times/week, 30 mins per session |
| Luo F 2021 | Conventional antihypertensive medication | Baduanjin exercise, 3 times/week, 40 mins per session |
| Zheng LW et al. 2021 | Lercanidipine tablets, 10mg qd; losartan potassium tablets, 50~100 mg qd | Baduanjin exercise, 5 times/week, 30 mins per session |
| Chen XX & Lv HQ 2006 | Nifedipine sustained-release tablets, 50~100 mg qd | Tai Chi exercise, once-daily, 40 mins per session |
