# Supplementary material for: Effects of different traditional Chinese exercise in the treatment of essential hypertension: a systematic review and network meta-analysis
Source: Front Cardiovasc Med. 2024 Feb 28;11:1300319. doi: 10.3389/fcvm.2024.1300319 (PMC10935740; doi:10.3389/fcvm.2024.1300319)
Supplement: Supplementary file 1 [file Datasheet1.zip › Supplementary material 5.docx]

**Supplementary material 5**

A total of 29 [1-29] studies reported specific treatment courses, the results of the meta-regression analysis indicated that while the course of treatment might not be the primary factor influencing the therapeutic effect, the results of meta-regression with treatment courses as covariate are shown in Table 1 and Table 2.

Table 1. Meta-regression analysis of SBP with treatment courses as covariate

|  | 2.5% | 25% | 50% | 75% | 97.5% |
| --- | --- | --- | --- | --- | --- |
| d.C.C+TC | 5.323 | 8.6 | 10.214 | 11.834 | 15.077 |
| d.C.C+BDJ | 2.007 | 5.693 | 7.518 | 9.347 | 13.017 |
| d.C.C+LZJ | -5.199 | 2.963 | 7.049 | 11.116 | 19.256 |
| d.C.C+WQX | -2.100 | 5.914 | 9.905 | 13.922 | 21.984 |
| d.C+TC.C+AE | -18.068 | -12.146 | -9.241 | -6.302 | -0.407 |
| sd.d | 6.110 | 7.35 | 8.164 | 9.116 | 11.479 |
| **B** | **-7.842** | **-3.002** | **-0.476** | **1.957** | **6.526** |

Table 2. Meta-regression analysis of DBP with treatment courses as covariate

|  | 2.5% | 25% | 50% | 75% | 97.5% |
| --- | --- | --- | --- | --- | --- |
| d.C.C+TC | 2.480 | 4.83 | 5.996 | 7.158 | 9.495 |
| d.C.C+BDJ | 2.831 | 5.535 | 6.878 | 8.217 | 10.908 |
| d.C.C+LZJ | -1.164 | 4.943 | 7.913 | 10.929 | 16.952 |
| d.C.C+WQX | 1.487 | 7.413 | 10.370 | 13.303 | 19.243 |
| d.C+TC.C+AE | -9.450 | -5.141 | -3.006 | -0.879 | 3.418 |
| sd.d | 4.439 | 5.364 | 5.961 | 6.658 | 8.391 |
| **B** | **-5.426** | **-2.004** | **-0.239** | **1.502** | **5.084** |

A total of 25 [1-9,11-23,27-29] studies reported specific treatment frequency, the results of the meta-regression analysis indicated that the varying frequency of TCEs may affect the antihypertensive effect, the results of meta-regression with frequency as covariate are shown in Table 3 and Table 4.

Table 3. Meta-regression analysis of SBP with frequency as covariate

|  | 2.50% | 25% | 50% | 75% | 97.50% |
| --- | --- | --- | --- | --- | --- |
| d.C.C+TC | 5.623 | 9.233 | 10.989 | 12.754 | 16.352 |
| d.C.C+BDJ | 1.981 | 5.494 | 7.208 | 8.938 | 12.473 |
| d.C.C+LZJ | -1.599 | 6.575 | 10.537 | 14.462 | 22.555 |
| d.C+TC.C+AE | -18.513 | -12.698 | -9.869 | -7.018 | -1.232 |
| sd.d | 5.681 | 6.98 | 7.833 | 8.855 | 11.425 |
| B | 0.076 | 6.081 | 8.981 | 11.839 | 17.548 |

Table 4. Meta-regression analysis of DBP with frequency as covariate

|  | 2.50% | 25% | 50% | 75% | 97.50% |
| --- | --- | --- | --- | --- | --- |
| d.C.C+TC | 2.550 | 5.074 | 6.305 | 7.531 | 10.037 |
| d.C.C+BDJ | 2.750 | 5.259 | 6.505 | 7.752 | 10.286 |
| d.C.C+LZJ | 2.101 | 7.954 | 10.802 | 13.636 | 19.429 |
| d.C+TC.C+AE | -9.489 | -5.403 | -3.388 | -1.371 | 2.705 |
| sd.d | 4.026 | 4.946 | 5.554 | 6.281 | 8.149 |
| B | 1.336 | 5.587 | 7.589 | 9.619 | 13.695 |

**Reference**

1. Xu H. Effect of 24-type simplified Tai Ji on blood pressure and quality of life in patients with hypertension. Hubei Journal of Traditional Chinese Medicine. (2016) 38(07):38-39.

2. Wang XB, Ye LP. Effect of 24-type simplified Tai Ji on primary hypertension with mild anxiety in the elderly. Fujian Journal of Traditional Chinese Medicine. (2019) 50(04):73-75. doi:10.13260/j.cnki.jfjtcm.011881

3. Jiang YH, Fu GL, Wang QP, Bao XP, Liu XX. Effect of Baduanjin on aged patients with hypertension and anxiety of liver yang hyperactivity syndrome. Journal of Qilu Nursing. (2019) 25(19):104-106. doi:10.3969/j.issn.1006-7256.2019.19.037

4. Zheng LW, Fan WY, Zhou LY, Chen F, Rao YL, Fang XH. Effect of Baduanjin in elderly hypertensive patients with frailty. Journal of Nursing Science. (2021) 36(24):90-93+97. doi:10.3969/j.issn.1006-7256.2019.19.037

5. Wang F, Wang H. A random controlled trial of Baduanjin combined with antihypertensive drugs in the treatment of I-grade hyperactivity of liver yang in hypertension. Special Health. 2021;(35):103-104.

6. Bai Y, Li XY, Xu WJ, Wei QY. Efficacy of Baduanjin combined with diltiazem hydrochloride sustained-release capsule in the treatment of grade Ⅰ-Ⅱ primary hypertension. Chinese Journal of Convalescent Medicine. (2020) 29(01):46-47. doi:10.13517/j.cnki.ccm.2020.01.018

7. Fan WY, Zheng LW, Chen F, Zhou LY, Rao YL, Fang XH. Effect of Baduanjin exercise on anxiety and depression in 38 elderly patients with essential hypertension. Fujian Journal of Traditional Chinese Medicine. (2021) 52(02):11-13. doi:10.13260/j.cnki.jfjtcm.012171

8. Zheng LW, Chen QY, Chen F, Mei LJ, Zheng JX. The influence of Baduanjin exercise on vascular endothelium function in old patients with hypertension grade 1. Chinese Journal of Rehabilitation Medicine. (2014) 29(03):223-227. doi:10.3969/j.issn.1001-1242.2014.03.006

9. Chen LH. Application of Baduanjin in rehabilitation nursing of elderly patients with hypertension. Journal of frontiers of medicine. (2016) 6(22):340-341.

10. Tang QH. Effects of traditional sports on the clinical symptom of aged intellectual patients with essential hypertension. Journal of Beijing Sport University. (2009) 32(02):67-69. doi:10.19582/j.cnki.11-3785/g8.2009.02.017

11. Lin Q, Yan XH. Promoting effect of body-building Baduanjin on rehabilitation of elderly patients with hypertension. Chinese Journal of Gerontology. (2017) 37(12):3024-3026. doi:10.3969/j.issn.1005-9202.2017.12.075

12. Dong DG, Yu ZD, Yu ZS. Effects of fitness Qigong Baduanjin on phlegm-dampness hypertension. Chinese Journal of Applied Physiology. (2020) 36(02):157-160. doi:doi.org/10.12047/j.cjap.5924.2020.035

13. Yang G. (2017) Effect of health Qigong·six-character formula on essential hypertension for liver-fire hyperactivity syndrome. [dissertation/master’s thesis]. [Henan]: Henan University of Chinese Medicine.

14. Liu J. Study on intervention of essential hypertension group by Tai Chi combined with health management. Guiding Journal of Traditional Chinese Medicine and Pharmacy. (2017) 23(05):64-66. doi:10.13862/j.cnki.cn43-1446/r.2017.05.021

15. Luo H. Clinical study on primary hypertension treated with Tai Ji combined with medication. China Medical Herald. (2006) (33):43-44. doi:10.3969/j.issn.1673-7210.2006.33.019

16. Liu T, Huang QD, Liu WZ. Effect of Tai Ji on blood pressure, hemorheology and long-term quality of life in elderly patients with hypertension. Chinese Journal of Gerontology. (2018) 38(06):1396-1398. doi:10.3969/j.issn.1005-9202.2018.06.050

17. Feng LJ, Guan L, Zhang DL, Lin ZC, Li MX, Jin RJ. Clinical effect of 24-simpilified Taijiquan exercise on the antihypertensive effect of elderly patients with essential hypertension and on their blood lipid level. Chinese Journal of Convalescent Medicine. (2018) 27(10):1009-1013. doi:10.13517/j.cnki.ccm.2018.10.001

18. Yang H, Zhao YN, Li JM. Effect of Baduanjin exercise on autonomic nerve reaction in patients with hypertension. Chinese Journal of Coal Industry Medicine. (2014) 17(07):1143-1146. doi:10.11723/mtgyyx 1007-9564 201407038

19. Chen H, Zhou YN. Effect of Baduanjin on blood pressure and serum high-sensitivity C-reactive protein in patients with essential hypertension. Chinese Journal of Rehabilitation Medicine. (2012) 27(02):178-179. doi:10.3969/j.issn.1001-1242.2012.02.023

20. Liang YH, Liao SQ, Han CL, Wang H, Peng Y. Effect of Baduanjin exercise intervention on blood pressure and blood lipid in patients with essential hypertension. Henan Traditional Chinese Medicine. (2014) 34(12):2380-2381. doi:10.16367/j.issn.1003-5028.2014.12.102

21. Yang XW. (2018) The effect of Tai Chi on sleep quality in elderly patients with essential hypertension. [dissertation/master’s thesis]. [Chengdu]: Chengdu University of Traditional Chinese Medicine.

22. Zhang XD, Deng B, Peng ZJ. Clinical Observation on Liuzjue Adjusting Breath Method in the Treatment of Hypertension Complicated with Anxiety and Depression. Chinese Medicine Modern Distance Education of China. (2022) 20(23):37-40. doi:10.3969/j.issn.1672-2779.2022.23.015

23. Chen FZ, Lv QB. Effect of Tai Ji Chuan on blood pressure in patients with hypertension. Modern Nurse. (2013) (4):18-19.

24. Lin H, Huang JS. Promoting effect of fitness Qigong Wuqinxi on rehabilitation of elderly patients with hypertension. Chinese Journal of Gerontology. (2013) 33(07):1645-1647. doi:10.3969/j.issn.1005-9202.2013.07.073

25. Tan LH, Jiang P, Ye L. Clinical study on eight-section brocade in adjuvant treatment of essential hypertension complicated with insomnia in senile patients. New Chinese Medicine. (2022) 54(16):175-178. doi:10.13457/j.cnki.jncm.2022.16.038

26. Zhou HH. Effect of wuqinxi on patients with essential hypertension. Medical Diet and Health. (2022) 20(11):186-188+192.

27. Luo F. Intervention effect and mechanism analysis of Baduanjin on elderly patients with type 2 diabetes mellitus complicated with hypertension. Chinese Journal of Geriatric Care. (2021) 19(05):13-16. doi:10.3969/j.issn.1672-2671.2021.05.005

28. Zheng LW, Chen ZS, Chen F, Liu J, Ge L. Mechanism of Baduanjin Exercise on Essential Hypertension Based on L-Arg/NOS/NO Pathway. Fujian Journal of Traditional Chinese Medicine. 2021;52(01):8-12. doi:10.13260/j.cnki.jfjtcm.012151

29. Chen XX, LV HQ. Effects of Taijiquan Exercise on Hypertension Patients'NO Consistency in Plasma,the Activity of RBC Na -K+.ATPase and Ca+-Mg+ATPase. Journal of Beijing Sport University. (2006) (10):1359-1361. doi:10.19582/j.cnki.11-3785/g8.2006.10.023
