# Supplementary material for: Effects of different traditional Chinese exercise in the treatment of essential hypertension: a systematic review and network meta-analysis
Source: Front Cardiovasc Med. 2024 Feb 28;11:1300319. doi: 10.3389/fcvm.2024.1300319 (PMC10935740; doi:10.3389/fcvm.2024.1300319)
Supplement: Supplementary file 1 [file Datasheet1.zip › Supplementary material 6.docx]

**Supplementary material 6**

Certainty of evidence in improving SBP

| Comparison | Number of studies | Within-study bias | Reporting bias | Indirectness | Imprecision | Heterogeneity | Incoherence | Confidence rating | Reason(s) for downgrading |
| --- | --- | --- | --- | --- | --- | --- | --- | --- | --- |
| Mixed evidence | | | | | | | | | |
| C VS C+TC | 11 | Some concerns | Low risk | No concerns | No concerns | Major concerns | No concerns | Very low | Within-study bias, heterogeneity |
| C VS C+BDJ | 9 | Some concerns | Low risk | No concerns | No concerns | Major concerns | No concerns | Very low | Within-study bias, heterogeneity |
| C VS C+LZJ | 2 | Some concerns | Low risk | No concerns | Major concerns | No concerns | No concerns | Very low | Within-study bias, imprecision |
| C VS C+WQX | 2 | Some concerns | Low risk | No concerns | Some concerns | Some concerns | No concerns | Very low | Within-study bias, imprecision, heterogeneity |
| C+TC VS C+AE | 2 | Some concerns | Low risk | No concerns | No concerns | No concerns | No concerns | Moderate | Within-study bias |
| C+BDJ VS C+AE | 3 | Some concerns | Low risk | No concerns | Some concerns | No concerns | No concerns | low | Within-study bias, imprecision |
| Indirect evidence | | | | | | | | | |
| C VS C+AE | 0 | Some concerns | Low risk | No concerns | Major concerns | No concerns | No concerns | Very low | Within-study bias, imprecision |
| C+TC VS C+BDJ | 0 | Some concerns | Low risk | No concerns | Major concerns | No concerns | No concerns | Very low | Within-study bias, imprecision |
| C+TC VS C+LZJ | 0 | Some concerns | Low risk | No concerns | Major concerns | No concerns | No concerns | Very low | Within-study bias, imprecision |
| C+TC VS C+WQX | 0 | Some concerns | Low risk | No concerns | Major concerns | No concerns | No concerns | Very low | Within-study bias, imprecision |
| C+BDJ VS C+LZJ | 0 | Some concerns | Low risk | No concerns | Major concerns | No concerns | No concerns | Very low | Within-study bias, imprecision |
| C+BDJ VS C+WQX | 0 | Some concerns | Low risk | No concerns | Major concerns | No concerns | No concerns | Very low | Within-study bias, imprecision |
| C+LZJ VS C+WQX | 0 | Some concerns | Low risk | No concerns | Major concerns | No concerns | No concerns | Very low | Within-study bias, imprecision |
| C+LZJ VS C+AE | 0 | Some concerns | Low risk | No concerns | Major concerns | No concerns | No concerns | Very low | Within-study bias, imprecision |
| C+WQX VS C+AE | 0 | Some concerns | Low risk | No concerns | Major concerns | No concerns | No concerns | Very low | Within-study bias, imprecision |

Certainty of evidence in improving DBP

| Comparison | Number of studies | Within-study bias | Reporting bias | Indirectness | Imprecision | Heterogeneity | Incoherence | Confidence rating | Reason(s) for downgrading |
| --- | --- | --- | --- | --- | --- | --- | --- | --- | --- |
| Mixed evidence | | | | | | | | | |
| C VS C+TC | 11 | Some concerns | Low risk | Low risk | No concerns | Some concerns | No concerns | Very low | Within-study bias, heterogeneity |
| C VS C+BDJ | 9 | Some concerns | Low risk | Low risk | No concerns | Some concerns | No concerns | Very low | Within-study bias, heterogeneity |
| C VS C+LZJ | 2 | Some concerns | Low risk | Low risk | No concerns | Major concerns | No concerns | Very low | Within-study bias, heterogeneity |
| C VS C+WQX | 2 | Some concerns | Low risk | Low risk | No concerns | Major concerns | No concerns | Very low | Within-study bias, heterogeneity |
| C+TC VS C+AE | 2 | Some concerns | Low risk | Low risk | Major concerns | No concerns | No concerns | Very low | Within-study bias, imprecision |
| C+BDJ VS C+AE | 3 | Some concerns | Low risk | Low risk | Some concerns | No concerns | No concerns | low | Within-study bias, imprecision |
| Indirect evidence | | | | | | | | | |
| C VS C+AE | 0 | Some concerns | Low risk | Low risk | Major concerns | No concerns | No concerns | Very low | Within-study bias, imprecision |
| C+TC VS C+BDJ | 0 | Some concerns | Low risk | Low risk | Major concerns | No concerns | No concerns | Very low | Within-study bias, imprecision |
| C+TC VS C+LZJ | 0 | Some concerns | Low risk | Low risk | Major concerns | No concerns | No concerns | Very low | Within-study bias, imprecision |
| C+TC VS C+WQX | 0 | Some concerns | Low risk | Low risk | Major concerns | No concerns | No concerns | Very low | Within-study bias, imprecision |
| C+BDJ VS C+LZJ | 0 | Some concerns | Low risk | Low risk | Major concerns | No concerns | No concerns | Very low | Within-study bias, imprecision |
| C+BDJ VS C+WQX | 0 | Some concerns | Low risk | Low risk | Major concerns | No concerns | No concerns | Very low | Within-study bias, imprecision |
| C+LZJ VS C+WQX | 0 | Some concerns | Low risk | Low risk | Major concerns | No concerns | No concerns | Very low | Within-study bias, imprecision |
| C+LZJ VS C+AE | 0 | Some concerns | Low risk | Low risk | Major concerns | No concerns | No concerns | Very low | Within-study bias, imprecision |
| C+WQX VS C+AE | 0 | Some concerns | Low risk | Low risk | Some concerns | Some concerns | No concerns | Very low | Within-study bias, imprecision, heterogeneity |
