# Supplementary material for: Effects of different traditional Chinese exercise in the treatment of essential hypertension: a systematic review and network meta-analysis
Source: Front Cardiovasc Med. 2024 Feb 28;11:1300319. doi: 10.3389/fcvm.2024.1300319 (PMC10935740; doi:10.3389/fcvm.2024.1300319)
Supplement: Supplementary file 1 [file Datasheet1.zip › Supplementary material 7.docx]

**Supplementary material 7**


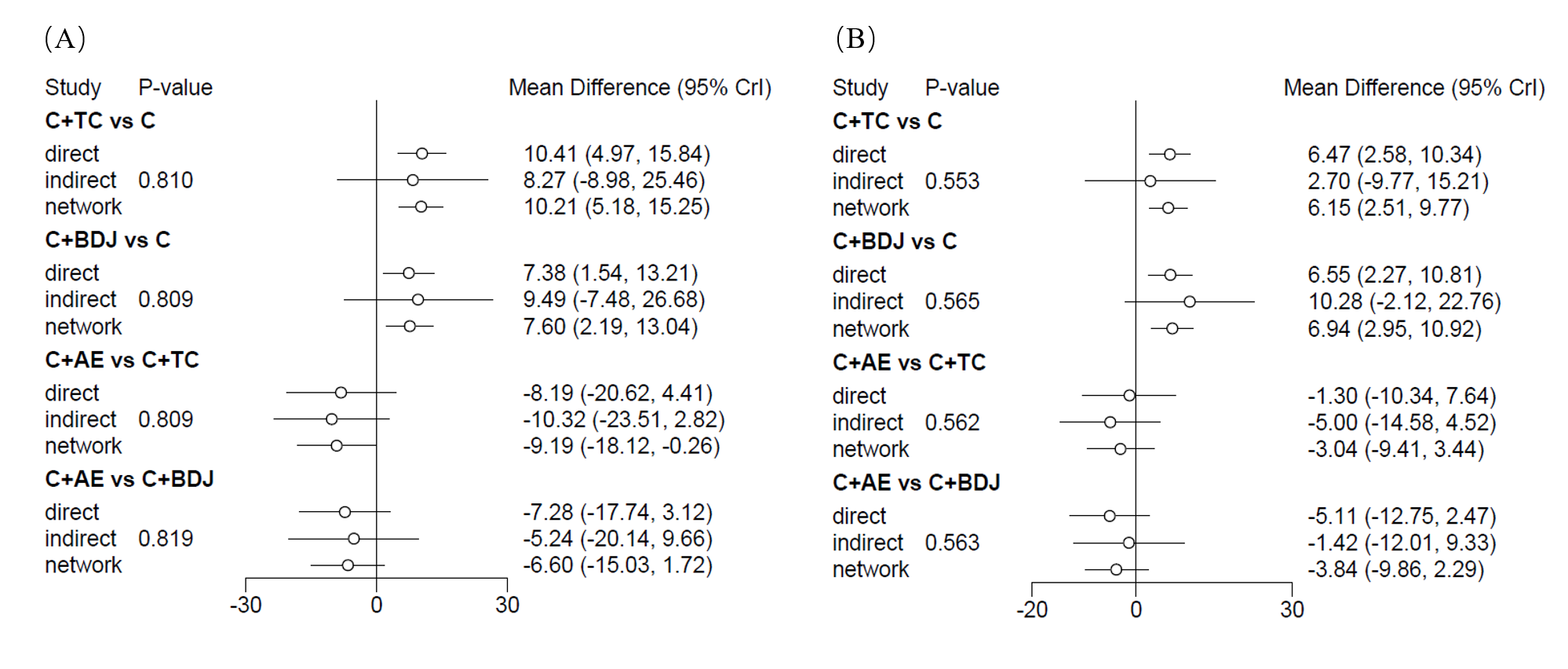


Fig .1. Node splitting analysis. (A) Node splitting results of SBP; (B) Node splitting results of DBP.


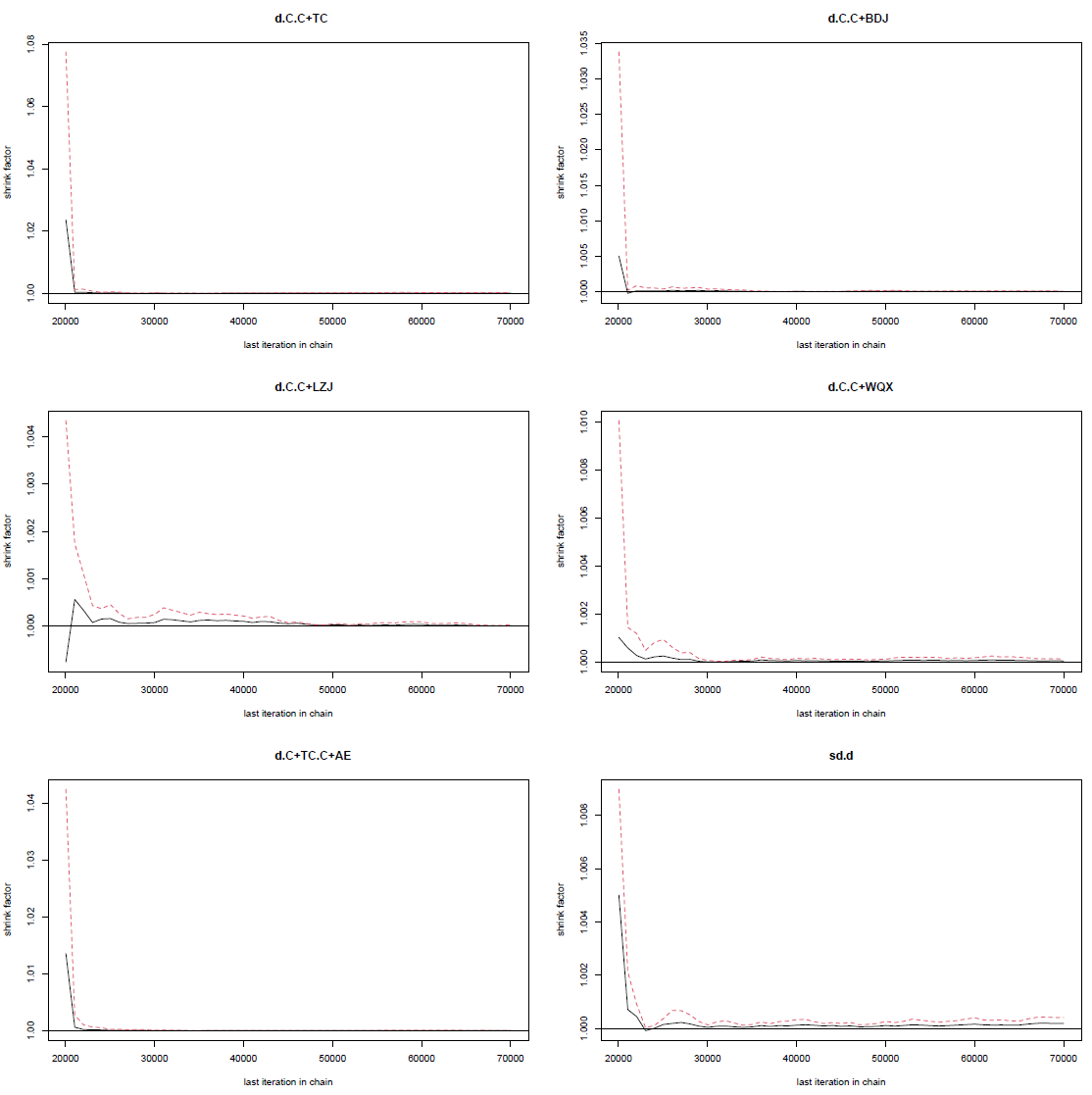


Fig .2. The diagnostic convergence results of SBP


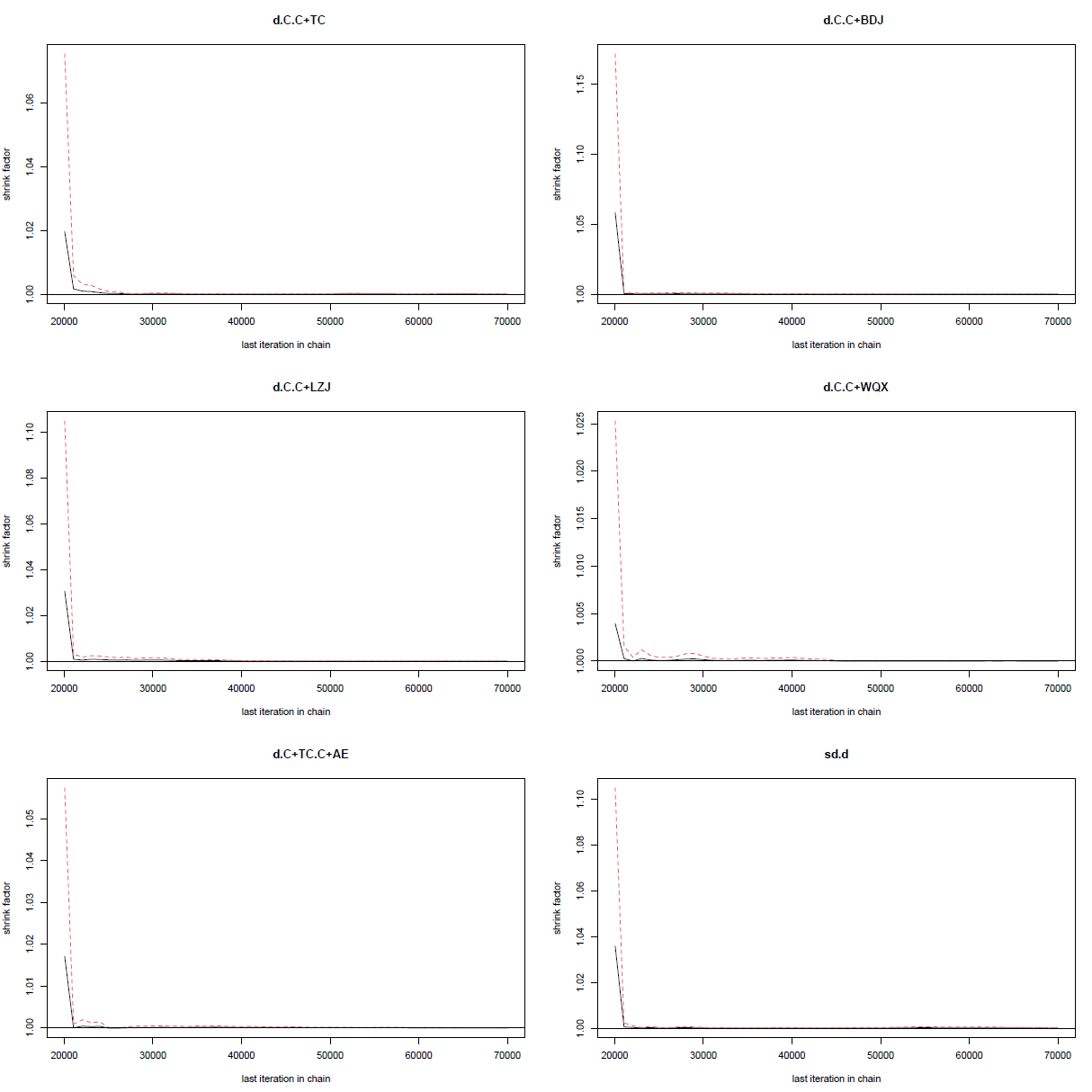


Fig .3. The diagnostic convergence results of DBP.
