# Supplementary figures and images for: Effects of different traditional Chinese exercise in the treatment of essential hypertension: a systematic review and network meta-analysis
Source: Front Cardiovasc Med. 2024 Feb 28;11:1300319. doi: 10.3389/fcvm.2024.1300319 (PMC10935740; doi:10.3389/fcvm.2024.1300319)

**Supplementary material 4**


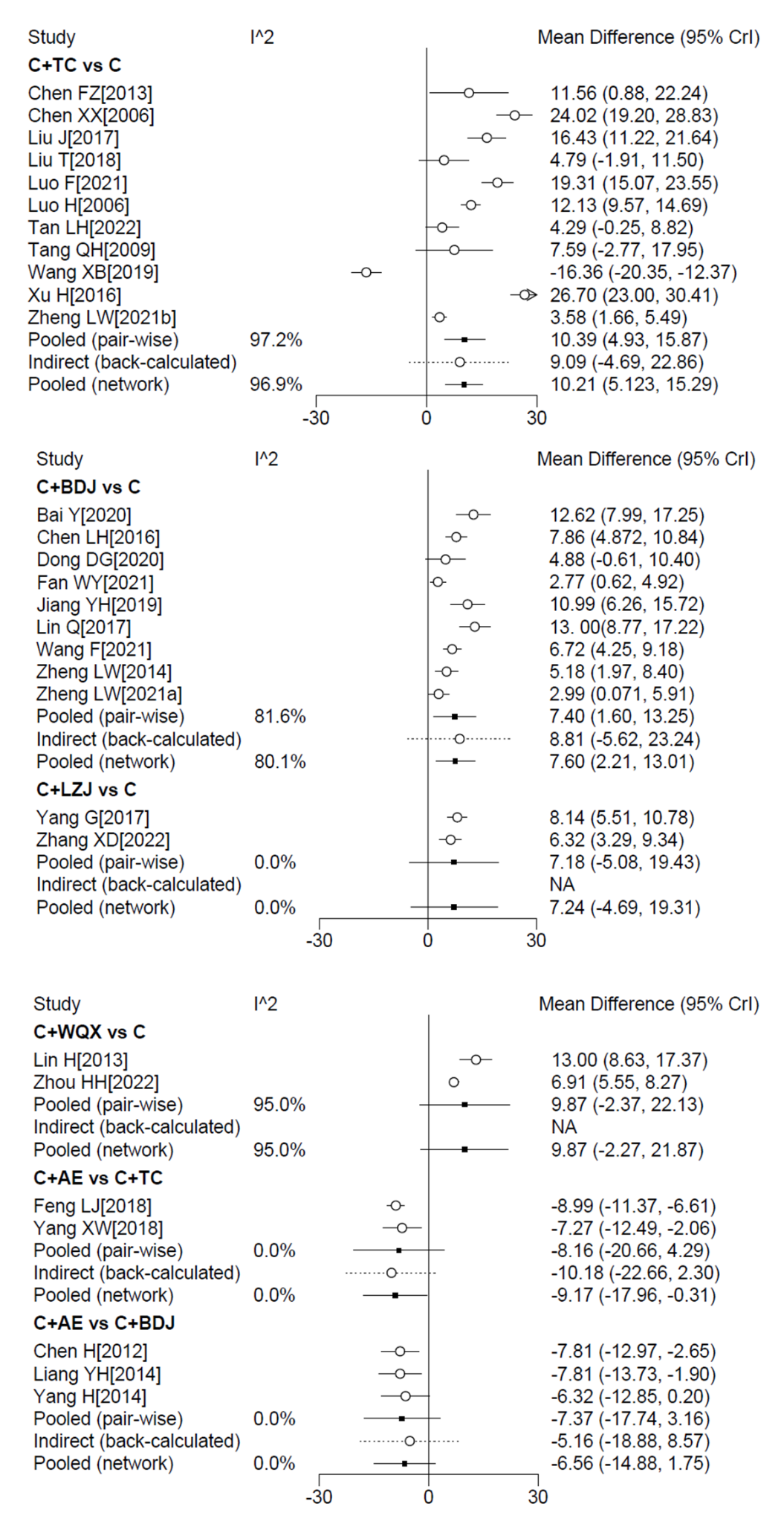


Fig.1. Heterogeneity test for SBP


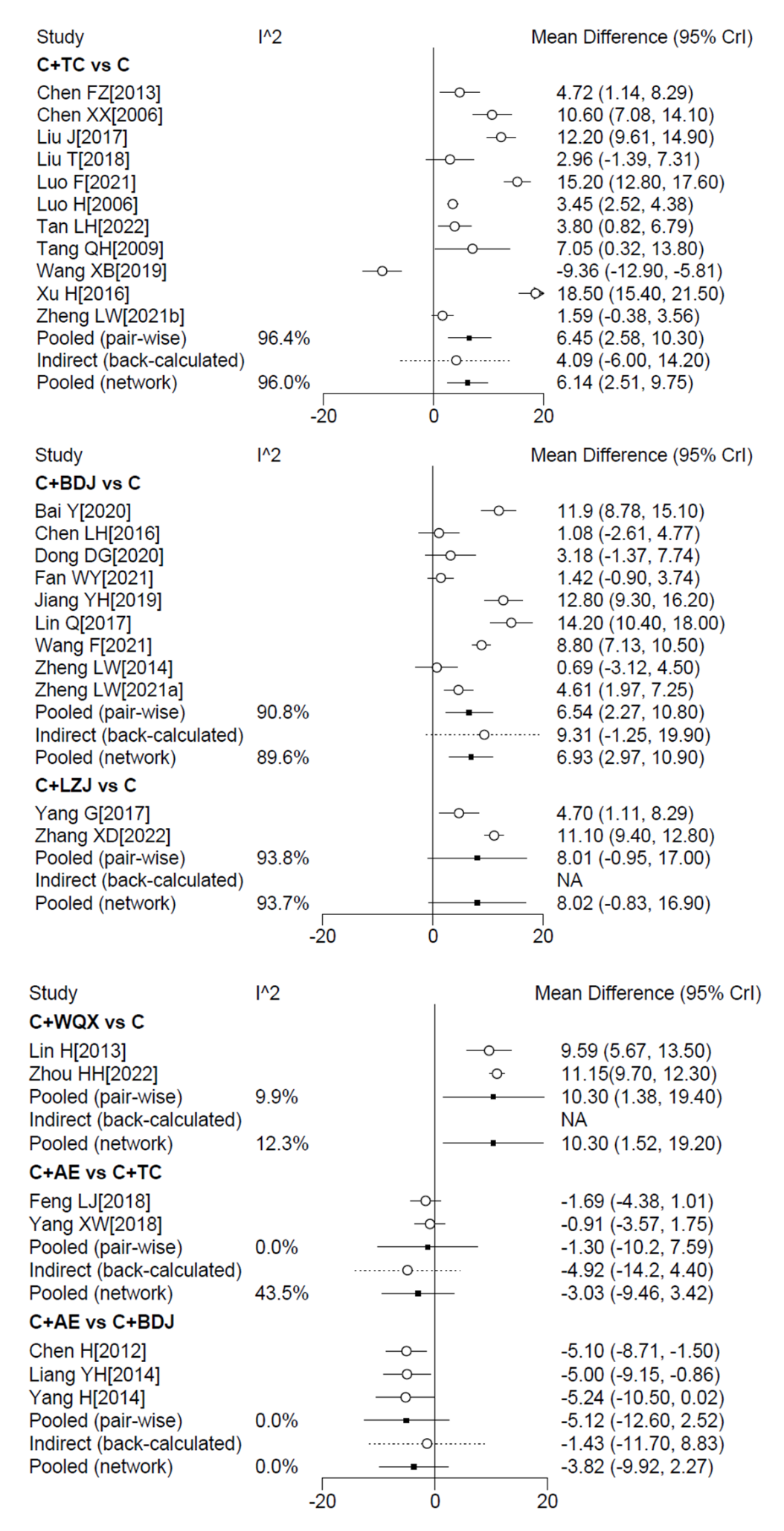


Fig.2. Heterogeneity test for DBP

Supplement: Supplementary file 1 [file Datasheet1.zip › Supplementary material 4.docx]

**Supplementary material 8**


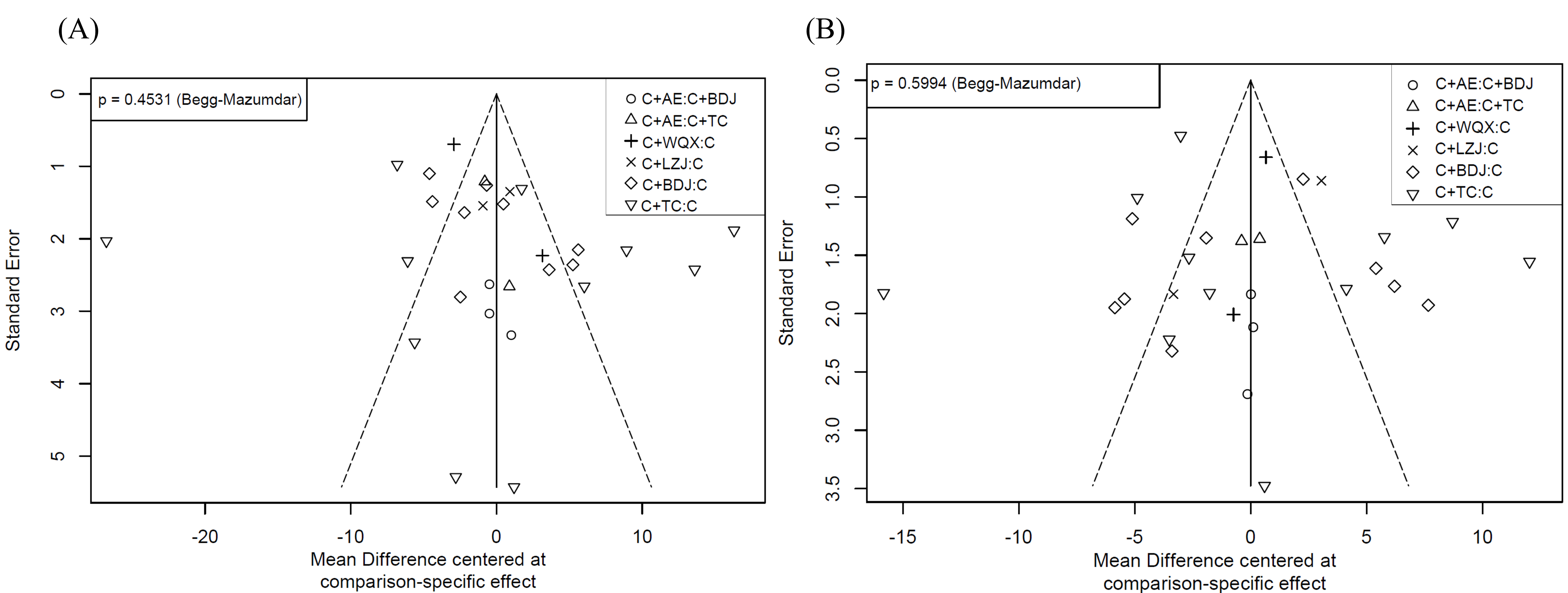


**Fig. S1**. (A) Begg’s test for SBP; (B) Begg’s test for DBP.

Supplement: Supplementary file 1 [file Datasheet1.zip › Supplementary material 8.docx]
